# Vick's Floral Guide for 1877

**Published:** 1877-02

**Authors:** 


*Vick's Floral Guide for* 1877, and *Vick's Illustrated Catalogue for* 1877, have been rece ved. This number of the "Guide" contains a very beautiful frontispiece entitled " Summer Bouquet," and is otherwise finely illustrated. So many are now interested in horticulture, that these publications of Mr. Vick must prove very acceptable.

His Flower and Vegetable Seed cannot be surpassed, as experience proves to us. The Floral Guide is a beautiful Quarterly Journal in German and English. Price 25 cents a year. The illustrated Catalogue contains 300 illustrations. The Flower and Vegetable Garden in paper 50 cents; elegant cloth cover $1.00. This work contains 150 pages and six fine cromos of flowers. Publisher, James Vick, Rochester, N. Y.

